# Analysis of a viral metagenomic library from 200 m depth in Monterey Bay, California constructed by direct shotgun cloning

**DOI:** 10.1186/1743-422X-8-287

**Published:** 2011-06-09

**Authors:** Grieg F Steward, Christina M Preston

**Affiliations:** 1 Department of Oceanography, University of Hawaii at Manoa, 1000 Pope Road, Honolulu, HI 96822, Hawaii; 2 Monterey Bay Aquarium Research Institute, 7700 Sandholt Road, Moss Landing, CA 95039-9644, USA

## Abstract

**Background:**

Viruses have a profound influence on both the ecology and evolution of marine plankton, but the genetic diversity of viral assemblages, particularly those in deeper ocean waters, remains poorly described. Here we report on the construction and analysis of a viral metagenome prepared from below the euphotic zone in a temperate, eutrophic bay of coastal California.

**Methods:**

We purified viruses from approximately one cubic meter of seawater collected from 200m depth in Monterey Bay, CA. DNA was extracted from the virus fraction, sheared, and cloned with no prior amplification into a plasmid vector and propagated in *E. coli *to produce the MBv200m library. Random clones were sequenced by the Sanger method. Sequences were assembled then compared to sequences in GenBank and to other viral metagenomic libraries using BLAST analyses.

**Results:**

Only 26% of the 881 sequences remaining after assembly had significant (E ≤ 0.001) BLAST hits to sequences in the GenBank nr database, with most being matches to bacteria (15%) and viruses (8%). When BLAST analysis included environmental sequences, 74% of sequences in the MBv200m library had a significant match. Most of these hits (70%) were to microbial metagenome sequences and only 0.7% were to sequences from viral metagenomes. Of the 121 sequences with a significant hit to a known virus, 94% matched bacteriophages (Families *Podo*-, *Sipho*-, and *Myoviridae*) and 6% matched viruses of eukaryotes in the Family *Phycodnaviridae *(5 sequences) or the Mimivirus (2 sequences). The largest percentages of hits to viral genes of known function were to those involved in DNA modification (25%) or structural genes (17%). Based on reciprocal BLAST analyses, the MBv200m library appeared to be most similar to viral metagenomes from two other bays and least similar to a viral metagenome from the Arctic Ocean.

**Conclusions:**

Direct cloning of DNA from diverse marine viruses was feasible and resulted in a distribution of virus types and functional genes at depth that differed in detail, but were broadly similar to those found in surface marine waters. Targeted viral analyses are useful for identifying those components of the greater marine metagenome that circulate in the subcellular size fraction.

## Introduction

Marine viruses are a source of enormous genetic diversity in the sea [1,2]. Having no inherent metabolic activity, viruses must interact with the replication machinery of their host organisms. As a by-product of these intimate intracellular interactions, viruses are a major driver of evolutionary change for cellular life [3]. Although viruses can provide significant benefits to their hosts [4,5], they are also a source of mortality for marine plankton and thus affect ecology and evolutionary selection [6]. Access to sequence information harbored in environmental viral assemblages has become of interest, because it provides insight into the types of viruses present in different habitats, and reveals the wealth of extracellular genetic information with which planktonic organisms are in constant communication [7].

Shotgun libraries have been constructed and analyzed that target marine viruses that are part of the plankton [8-13], the benthos [14,15], or are associated with marine life [16,17]. Common themes that have emerged from such analyses are that the diversity of marine viruses is enormous, that the functions of the majority of sequences derived from marine viral metagenomes are unknown [18], and that a large proportion of the DNA-containing viruses infect prokaryotes [2], while most RNA-containing viruses infect eukaryotes [7,19].

Here, we report an analysis of a shotgun library prepared from DNA extracted from a purified viral assemblage harvested in the epipelagic-mesopelagic boundary in Monterey Bay, California. Unlike all previous metagenomes that have specifically targeted viruses, this library was produced with no prior *in vitro *amplification and appears to be the first reported for seawater collected on one occasion and from a single depth below the euphotic zone.

## Materials and methods

### Collection and Purification of Viruses

**Figure 1:**
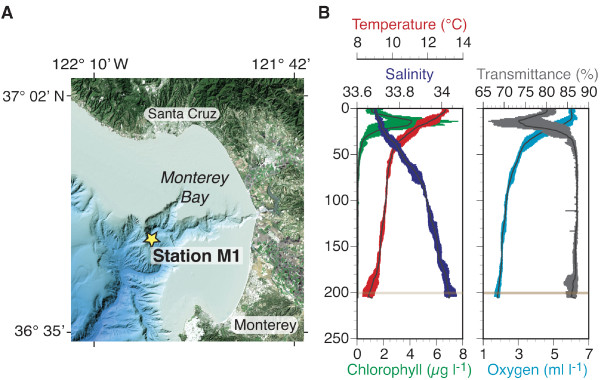
**Sampling location and hydrographic properties at the time of collection**. Panel A: The location of Station M1 in Monterey Bay, CA. Panel B: Profiles of hydrographic properties from the ten casts with an instrument suite and Niskin bottle rosette with which water and data were collected. Data for temperature, salinity, fluorescence-based chlorophyll concentration, dissolved oxygen, and transmissivity at each depth are plotted as the mean (black lines) and range (shown in color) for the ten casts. The horizontal line at 200 dbar highlights the depth at which water was collected.

Seawater from a depth of ca. 200 m (200 ± 1 dbar) was collected from ten casts of a Niskin bottle rosette on July 25, 2001 at Station M1 (36.75° N Latitude, 122.03° W Longitude) in Monterey Bay, CA, USA. The station is located at the mouth of the bay over an undersea canyon with a total water depth of ca. 1000 m (Figure 1A). A suite of sensors on the sampling rosette provided profiles (Figure 1B) of temperature and salinity (Sea-Bird 911*plus*), chlorophyll fluorescence (WET Labs Flash Lamp Fluorometer), dissolved oxygen (Sea-Bird SBE 13), light transmission (WET Labs C-star). At the depth of collection, temperature ranged from 8.3 to 9.0°C and salinity from 34.01 to 34.07, depending on the cast. Approximately 1,190 liters of seawater were filtered through 30 μm nylon mesh filter, and plankton in the filtrate were concentrated to 415 ml final volume by tangential flow ultrafiltration using an Amicon model DC-10L system (Millipore, Billerica, Massachusetts) with a 30,000 Da nominal molecular weight cut-off (30 kDa NMWCO) hollow fiber cartridge (H10P30-20, Millipore). The hollow fiber filter was subsequently back-flushed with 8 L of filtrate and the flush volume was recirculated then concentrated to 530 ml. The primary concentrate and subsequent wash were pooled and further concentrated to 33 ml using a Pellicon XL50 system (Millipore) with a 30 kDa NMWCO cartridge (Pellicon 2 Maxi Filter, Millipore). The concentrate was centrifuged at 12,000 × g for 20 min to pellet prokaryotes and larger cells. The supernatant (containing predominantly viruses) was then preserved with sodium azide (0.5% final conc.) and stored at 4°C (5 weeks).

To remove any residual cells, the viral concentrate was filtered twice through a 0.2 μm syringe-tip filter (Acrodisc, Pall Gelman). Viruses in the remaining sample (ca. 32 ml) were further concentrated using a 30 kDa NMWCO centrifugal ultrafiltration device (Ultracel PL-30 membrane, Millipore) then washed by addition of 2 ml of 0.02 μm-filtered MSM followed by re-concentration. The final concentrate was recovered and the ultrafilter washed again with 500 μl of MSM. The concentrate and the wash were pooled and the resulting viral concentrate (1.6 ml) was stored at 4°C (three weeks) to await further purification in a density gradient.

Viruses in the concentrate were banded in a self-forming, CsCl equilibrium buoyant density gradient (1.5 g ml^-1 ^initial density) in a TLN-100 rotor at 55,000 rpm at 10°C for 48 hours. Twelve fractions (ca. 0.3 ml each) were collected and the density of each was calculated from volume and mass measurements using a micropipet and a microbalance [20]. Subsamples for determining the virus concentration in each fraction were diluted into 0.02 μm-filtered MSM (450 mM NaCl, 50 mM MgSO_4_, 50 mM Tris-HCl pH 8.0; Steward 2001), fixed with formaldehyde, and then processed and enumerated by epifluorescence microscopy using the SYBR Green I protocol (Noble and Fuhrman 1998).

### Extraction and Evaluation of Viral DNA

Four fractions from the CsCl gradient containing virus-like particles (VLPs) were pooled then concentrated in a 100 kDa NMWCO centrifugal ultrafiltration unit (Centricon 100, Millipore). CsCl and other low molecular weight solutes were removed by washing the concentrate two times with 0.5 ml molecular-biology grade TE buffer (Ambion, Austin, TX) according to the device manufacturer's instructions. The final concentrate volume in TE was approximately 150 μl to which was added 350 μl of sterile-filtered (0.22 μm Sterivex; Millipore) sucrose lysis buffer (50 mM Tris-HCl, pH 8.0, 40 mM EDTA, and 0.75 M Sucrose). The final concentrate plus lysis buffer was recovered from the ultrafiltration unit and stored at -20°C (two days).

The viral pool was thawed on ice and the volume brought up to 900 μl with additional sucrose lysis buffer. Proteinase K (0.5 mg ml^-1 ^final conc., Fisher Scientific, Pittsburgh, PA) and SDS (1% final conc., Fisher Scientific) were added followed by incubation at 55°C for 2 hours with agitation. The sample was extracted with an equal volume of phenol:chloroform:isoamyl alcohol (25:24:1, v:v:v, equilibrated with TE, pH 8.0, Sigma Aldrich, St. Louis, MO). The aqueous phase was then transferred to a Centricon 100, washed three times with 1 ml TE buffer as described above, then reduced to a minimal volume (ca. 10 μl), and stored at -80°C.

The purified DNA was analyzed by pulsed field gel electrophoresis (PFGE) using a CHEF DR II instrument (BioRad, Hercules, CA). For comparison, a subsample of the pooled CsCl gradient fractions was also prepared for PFGE using a previously described protocol (Steward 2001) and run on the same gel. In this latter case, viruses in the subsample were concentrated on a microcon filter and the retentate was rinsed twice with TE then recovered in a volume of ca. 30 μl. Loading buffer was added and the sample was heated to 60°C for 10 min, then cooled on ice. Size standards consisted of a 5 kb and lambda DNA ladders (BioRad). Samples and standards were analyzed on a 1% agarose gel run for 13 hours at 16°C under an applied voltage gradient of 6 V cm^-1 ^with switch interval ramping linearly from 1-5 seconds. The gel was post-stained with 0.5 μg ml^-1 ^of ethidium bromide and visualized on a FlourImager (Amersham, Piscataway, NJ).

To check for bacterial contamination of the viral fraction, the extracted viral DNA was screened for the presence of 16S rRNA genes by PCR using bacterial specific primers 27F and 1492R [21] as previously described [22]. The resulting product was ligated into the TA cloning vector 2.1 (Original TA Cloning Kit, Invitrogen, Carslbad, CA) and transformed into *E. coli *by heat shock of chemically competent cells following the manufacturer's instructions.

Restriction fragment length polymorphism (RFLP) analysis was performed on nine clones. One of the insert-containing clone was sequenced by dideoxynucleotide termination using BigDye Chemistry v.3.0 (Applied Biosystems, Foster City, CA) using the M13F and M13R primer sites on the cloning vector. Reactions were analyzed on an ABI 3100 genetic analyzer (Applied Biosystems).

### Library Construction and Sequencing

A viral shotgun library was then constructed using TOPO Shotgun Subcloning Kit version A (Invitrogen) according to the manufacturer's instructions. Briefly, ca. 6 μg of DNA was added to shearing buffer and passed through a nebulizer on ice for 90 seconds at 10 psi of compressed, filtered air. The DNA (in 700 μl of buffer) was then precipitated with an equal volume of isopropanol after addition of sodium acetate (0.3 M final), and glycogen (80 μg) as a co-precipitant [23]. Precipitated DNA was washed once with 70% ethanol and the dried pellet resuspended in 24 μl of water. The DNA was repaired to produce blunt ends with T4 and Klenow DNA polymerases, dephosphorylated with calf intestinal phosphatase, then precipitated in ethanol [23]. Half of the DNA was then cloned into the pCR4Blunt-TOPO vector (Invitrogen). The ligation reaction was desalted by drop dialysis on a 0.025 μm pore size mixed cellulose ester membrane (Millipore) floating on 0.5× TE buffer for 1 hour. TOP10 electrocompetent cells (Invitrogen) were transformed with the recombinant DNA by electroporation. Colonies were arrayed into 96-well plates and stored at -80°C in LB amended with 50 μg/ml kanamycin (Sigma) and glycerol (7% final conc, v:v).

Initial sequencing of clones from the first library (MBv200mA) revealed that many of the inserts were small, so a second library (MBv200mB) was constructed from the remaining sheared, blunt-end-repaired, dephosphorylated DNA as described above, but after size selection. For size selection, the DNA was separated by electrophoresis in a 1% low-melting-point agarose gel (NuSieve, Cambrex, Rockland, ME). DNA between 1.4 to 4 kb was excised from the gel and recovered with a S.N.A.P column (Invitrogen) following the manufacturer's instructions. Plasmid minipreps were prepared using the Montage Miniprep Kit (Millipore). The average insert size of the shotgun clones was determined by agarose gel electrophoresis of clones digested with the restriction enzyme *Eco*RI. Clones from the libraries were end-sequenced using dye terminator technology as described above.

### Bioinformatic Analyses

A total of 1,055 sequences (MBv200mA plus MBv200mB) were processed using the Sequencher software (GeneCodes Corporation) to remove vector and trim low quality sequence. Sequences were trimmed to a maximum of 500 bp and sequences less than 100 bp were discarded, leaving a total of 907 sequences for analysis. Sequences were assembled in Sequencher with the requirement of a minimum 21 bp overlap and 98% identity. Sequences were then compared to various nucleotide and protein databases using blastx and tblastx algorithms [version 2.2.18; [24]]. Sequences have been deposited in the Genome Survey Sequence Database of GenBank http://www.ncbi.nlm.nih.gov/dbGSS/, with accession numbers [GenBank:JJ725428 to JJ726334].

The tblastx algorithm was used to query the nucleotide collection (nr/nt), genomic survey sequences (GSS), and environmental sample (env_nt) databases downloaded from the National Center for Biotechnology Information (NCBI) on July 2008. The blastx algorithm was used to query the non-redundant protein sequences (nr), environmental samples (env_nr), and clusters of orthologous groups of proteins (COG) databases from NCBI and the Pfam [25] and KEGG [26] databases. BLAST results were parsed to save the top-scoring hits for each sequence. A Perl script was also run that extracted any hits to a sequence containing at least one following virus-related keywords: "phage" or "virus" (alone or as part of a longer word), "capsid", "tail", "integrase", "base plate", "baseplate", or "portal". All sequences in the automatically generated list were then inspected individually to verify that the hits identified were to sequences of viral origin. Information (database queried, sequence ID, description, E-value) on the top-scoring and keyword-containing hits for each sequence in each database were compiled in a spreadsheet program (Excel, Microsoft Corp.) and individually annotated to note the sources of the matching sequences (eukaryote, bacteria, archaea, virus, mobile element, microbial metagenome, viral metagenome). Sequences were also analyzed using MG-RAST [27], an online metagenome annotation service (http://metagenomics.anl.gov/),

We compared our library to seven other metagenomic libraries prepared from the viral fraction of seawater by BLAST analysis. Sequences from Mission Bay in San Diego, CA and Scripps Pier in La Jolla, CA [11], the Chesapeake Bay [9], and from the Sargasso Sea, Gulf of Mexico, Coastal British Columbia, and Arctic Ocean [10] were download from the NCBI FTP site on February 11, 2009. Each of these datasets was then compared to the MBv200m library using tblastx. Because of the asymmetric nature of BLAST, which was accentuated by the large disparities in numbers and lengths of sequences among libraries, we chose to conduct the BLAST analysis in a reciprocal manner: MBv200m as the query against each library and each library as the query against MBv200m, in each case we counted hits with E-value of ≤ 10^-5^. To handle the computationally intensive nature of BLAST and parsing tasks, a custom script was used, which uses the python SciPy library http://www.scipy.org/[28] and runs the jobs on a 64-node compute-cluster in an embarrassingly-parallel way (each sequence blasted and parsed independently).

Results of the BLAST data were used to calculate three parameters for each pair-wise library comparison: 1) the hits in MBv200m expressed as a percentage of the total sequences in MBv200m, 2) the hits in each other library expressed as a percentage of the sequences in that library, and 3) the reciprocal of the hits in MBv200m after normalizing to the total number of sequences in each query library.

Differences in read length are expected to affect the percentage of hits between two libraries [7]. To compensate for this, we calculated the same parameters as above, but after normalizing the number of sequences in each library to the mean read length of MBv200m (e.g., for a library with a mean read length half that of MBv200m, the length-normalized number of sequences in that library would be half of the total).

We also compared our library to six other marine viral metagenomes in MG-RAST. The Scripps Pier and Mission Bay libraries were not available in MG-RAST, but the other five viral metagenomes noted above were. To those we added a phage metagenome from Tampa Bay, FL [29].

This work did not involve experimentation on humans or animals.

## Results

### The Viral Concentrate

**Figure 2:**
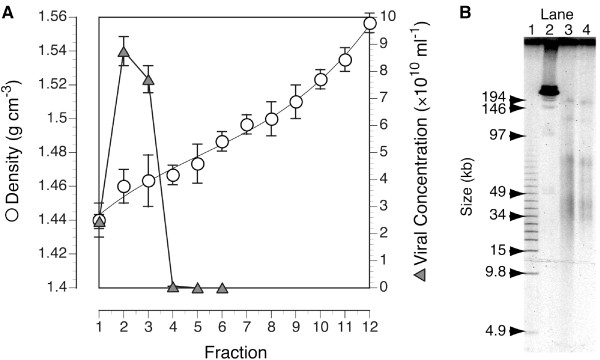
**Buoyant density and genome size distribution of virus-like particles used to construct the library**. Panel A: The density (open circles) of each fraction collected from an equilibrium buoyant density gradient is plotted along with the concentration of viruses (filled triangles) in the fraction. Fractions 1 through 4 from the top of the gradient were harvested and pooled to construct the library. Error bars for density represent the standard deviation from triplicate measurements. Error bars for viral abundance represent the standard deviation of counts from multiple fields of a single filter for each fraction. Panel B: Image of DNA from the purified viral fraction separated by pulsed-field gel electrophoresis. Lane 1, Size standard (5 kb ladder); Lane 2, Size standard (Lambda DNA ladder); Lane 3, pooled viruses loaded after simple heat shock in TE to release DNA; Lane 4, DNA from purified viruses after organic extraction with phenol-chloroform.

Epifluorescence microscopy of the CsCl continuous gradient fractions revealed that the VLPs in the targeted size range were concentrated in the top four fractions of the gradient, which had average buoyant densities ranging from 1.44-1.47 (Figure 2A). A total of 1.9 × 10^11 ^viral particles were recovered from the CsCl gradient fractions, which yielded 8 μg of DNA. Analysis of the sample by pulse field gel electrophoresis showed similar banding patterns from the phenol:chloroform extracted viral DNA and the viral DNA from the same sample prepared by simply heating in TE (Figure 2B). Four major size classes were observed, 30-45 kb, 60-80 kb, 125 kb and a greater than 146 kb fraction.

During counts of viruses in the CsCl gradient fractions, no particles that were obviously cells were observed, but PCR amplification of the extracted DNA using bacterial primers for 16S rRNA genes resulted in weak amplification. Analysis of nine clones revealed a single RFLP pattern indicating that the amplified product was dominated by a single bacterial rRNA gene type. The sequence of a representative clone was 98% similar (over 600 bp) to a psychrophilic marine bacterium PS03 [GenBank:AF200213].

### Library Analysis

Our first viral library (MBv200mA), prepared with sheared DNA that was not size selected, produced many clones with short inserts (< 200 bp). The average insert size of the second size-selected library (MBv200mB) was 1.9 kb. Sequences from these libraries were combined and treated as a single library (MBv200m). Assembly of the sequences resulted in 52 of the 907 sequences forming 26 contigs, each comprised of two sequences. Twenty of those were contigs formed from the forward and reverse read of the same clone. The remaining six contigs from 12 sequences (1.3% of the total) were formed from clones with different names (i.e., separate clone picks in different library locations).

### Sequence Analysis

**Figure 3:**
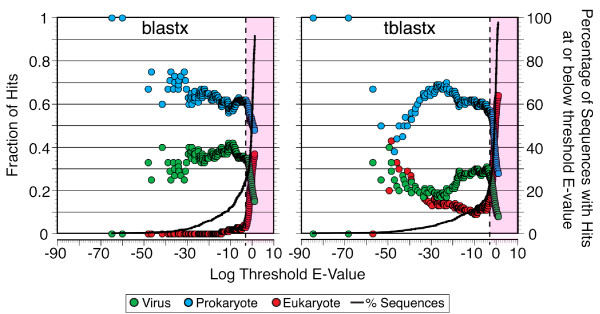
**Changes in the distribution of blastx and tblastx hits as a function of E-value threshold**. The proportion of top-scoring matches to viruses (including bacteriophages), prokaryotes, or eukaryotes is plotted as a function of the E-value threshold for blastx (left panel) or tblastx (right panel). The vertical dashed line represents the frequently used threshold of 10^-3^, beyond which hits would not be considered significant (pink shaded region). The solid line in each graph shows the cumulative percentage of sequences that lie at or below a given E-value threshold.

After assembly, the remaining 881 sequences (389,597 bases in total) were compared to sequence databases to identify the genes recovered. The distribution of hits to eukaryotes, prokaryotes, or viruses varied as a function of the threshold E-value and differed for blastx vs. tblastx (Figure 3). At the commonly used threshold of 10^-3^, the percentage of sequences with a hit to any of these three groups of organisms was similar (26% for blastx and 23% for tblastx), but the number of hits specifically to viral sequences was 1.6 times higher using blastx (72 vs. 45). The greater percentage of hits to viral sequences when using blastx was consistent across a broad range of threshold values, but in neither case did the viral hits exceed 42% (Figure 3). The lower proportion of hits to viruses with tblastx was compensated primarily by a higher proportion of hits to eukaryotes. From the plots of the hit distribution vs. threshold E-value, we observed sharp declines in the proportion of hits to viruses and prokaryotes between E-values of 10^-3 ^and 10^-2 ^for blastx and between 10^-4 ^and 10^-3 ^for tblastx (Figure 3), which was again compensated by an increasing proportion of hits to eukaryotes.

**Figure 4:**
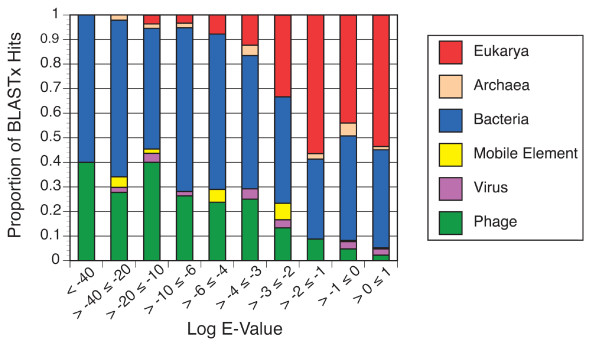
**Distribution of blastx hits in various E-value ranges**. Proportion of top-scoring blastx matches most similar to bacteriophages, eukaryotic viruses, mobile genetic elements, or members of the Domains *Bacteria*, *Archaea*, or *Eukarya *for different ranges of E-values.

A more detailed view of the blastx hit distributions in different E-value ranges (Figure 4) showed that the majority of hits are to bacteria and bacteriophages in all bins ≤ 10^-2^. Hits to archaea, eukaryotic viruses, and mobile genetic elements occurred throughout most of the E-value range, but were always minor contributors (≤ 10% combined) and their contributions did not vary systematically. In all bins having E-values ≤ 10^-3^, bacteriophages represented 24 to 40% of the hits. In each bin with E-values > 10^-3^, the proportion of hits to bacteriophages dropped by one-third to one-half relative to the preceding bin (Figure 4).

Analysis of MBv200 with MG-RAST v2 resulted in no significant hits to 16S rRNA sequences, but also no protein-based hits to viruses. When re-analyzed with the recently released MG-RAST version 3, 63 of 881 sequences (7.15%) had a significant match, and the majority of these (55.6%) were to the subsystem "Phages, Prophages, Transposable Elements, Plasmids". Within that category, 88.6% were to phages or prophages and the remainder to pathogenicity islands. The next most represented categories were "Nucleosides and Nucleotides" (9.5%), "DNA Metabolism" (8%) and "Protein Metabolism" (8%).

**Figure 5:**
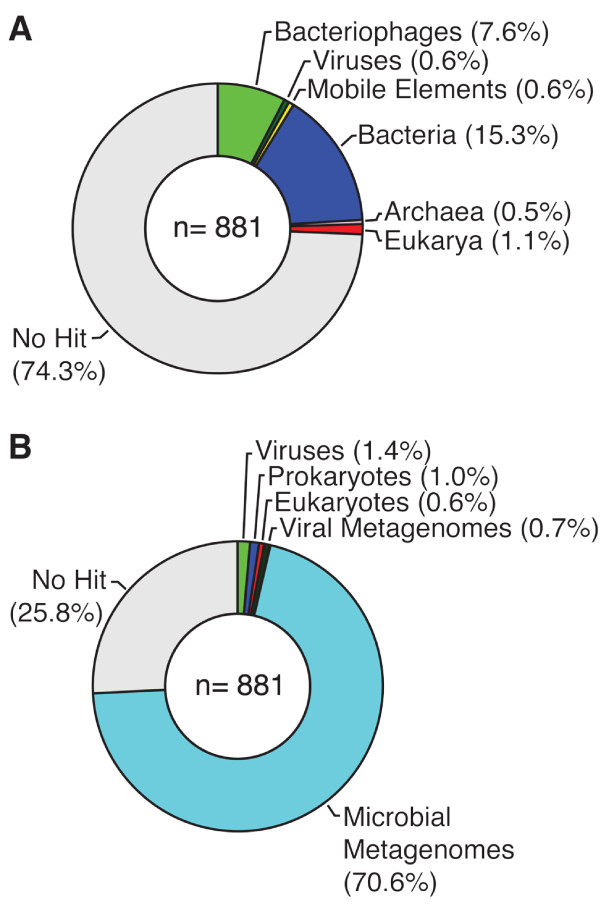
**Proportion of BLAST hits to sequences from various sources**. Donut charts illustrating the proportion of sequences in the Monterey Bay viral metagenome library that had significant (E-value ≤ 10^-3^), best blastx matches to bacteriophages, eukaryotic viruses, mobile genetic elements, or members of the Domains *Bacteria*, *Archaea*, or *Eukarya*, or which had no significant match (E-value > 10^-3^). Panel A: BLAST results when using only the GenBank nr database. Panel B: the distribution of top-scoring matches when sequence data from viral and microbial metagenomes were included in the analysis. In Panel B, eukaryotic viruses, bacteriophages, and mobile genetic elements were combined into the single category "Viruses" and bacteria and archaea were combined into the category "Prokaryotes".

Comparison of all sequences against the GenBank nr database using blastx resulted in 74% of the sequences having no significant hit (Figure 5A). Bacteriophages and viruses accounted for 8.2% of the top hits, other mobile elements accounted for 0.6% and hits to the members of the domains *Bacteria*, *Archaea *and *Eukarya *accounted for 15.3, 0.5, and 1.1%, respectively. Although a large number of sequences had no significant match to sequences of known phylogenetic affiliation in the GenBank nr database, the majority of them had top hits to sequences from metagenomic studies curated in the "Environmental (env)" and "Genome Survey Sequences (GSS)" section of GenBank (Figure 5B). Only 3.7% of the sequences had a better hit to a sequence in GenBank nr than to sequences from marine metagenomic studies. None of the sequences in the Monterey Bay library had significant similarity to a 16S rRNA gene.

**Figure 6:**
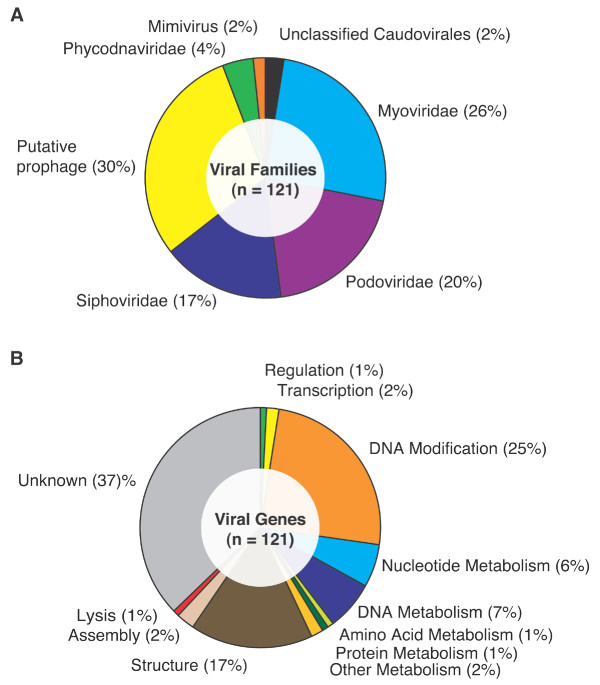
**Phylogenetic affiliations and functions of viral BLAST hits**. Panel A: Proportion of sequences recovered in the Monterey Bay 200 m viral metagenome with significant similarity to different virus types and Panel B: the proportion of different functions represented among those sequences.

Since top hits are not necessarily the best guide to the phylogenetic identity of a sequence, we also determined what proportion of the sequences had any significant hit to a virus sequence, even if it was not the top hit. In this case, just over half (51%) of all significant hits included a similarity to a viral sequence. A total of 143 sequences had a significant match to a bacteriophage, virus, or viral metagenome sequence. Excluding the hits to sequences from viral metagenomes, there remained 121 sequences with significant, but not necessarily best, matches to known bacteriophages or viruses. Of these, 94% were to sequences from bacteriophages and 6% were to eukaryotic viruses (Figure 6A). All of the bacteriophage matches were to members of the Order *Caudovirales *(tailed viruses; 66%) or to known or putative prophages (30%). There were similar proportions of matches to the Families *Myoviridae *(26%), *Podoviridae *(20%), and *Siphoviridae *(17%) that comprise the Order *Caudovirales*. The only eukaryotic hits were to members of the Family *Phycodnaviridae *(4%) and to the mimivirus (2%).

A known or putative function was noted for 63% of the bacteriophage or viral matches and 37% had unknown function (Figure 6B). Of those with an ascribed function, genes involved in DNA modification were the most prevalent (25%), followed by structural genes (17%). Other functions noted among the matches were gene regulation (1%), transcription (2%), nucleotide metabolism (6%), DNA metabolism (7%), amino acid metabolism (1%), protein metabolism (1%), other metabolism (2%), assembly (2%) and lysis (1%). Sixteen sequences had a significant hit to a terminase (nine of these were the top hit) and seven to portal proteins (six were the top hit). There were four significant matches each to tail fiber, integrase, helicase and ribonucleotide reductase genes, and three each to phage DNA polymerases and phage major capsid proteins.

### Comparison with other Viral Metagenomes

**Figure 7:**
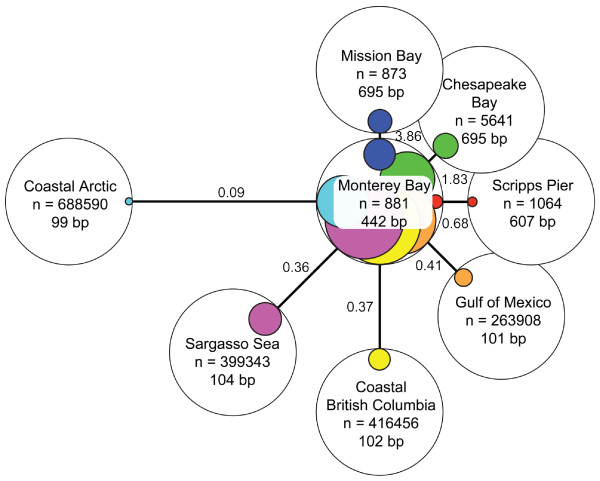
**Comparison of the MBv200 m library to other viral metagenomic libraries by reciprocal BLAST analyses**. Each library is represented as a white circle, in the center of which appears the library name, the number of sequences (un-normalized), and the average read length. The relative distances between each library and MBv200 m (solid connecting lines) were calculated by BLAST queries of each library against the Monterey Bay library to determine the number of hits per 100 read-length-normalized sequences in the query library. In each case, the normalized number of hits (numbers adjacent to the lines) was converted to a relative distance by taking its reciprocal. The areas of the white circles represent 100% of the sequences in each library and the areas of the colored circles within represent the read-length-normalized proportion of that library's sequences that had a significant hit when queried with the other library by BLAST.

Using pair-wise, reciprocal blast analysis, we found that the number of hits to the MBv200m library generally increased as a function of the query library size (ranging from a low of 0.8% for Scripps Pier to a high of 28% for Coastal British Columbia; Figure 7). An obvious outlier in this trend was the Arctic Ocean, which was the largest library, but had the third lowest percentage of hits (11.6%) to MBv200m. After normalizing for query library size and differences in sequence length, the libraries prepared from other bays (Mission Bay, San Diego, CA, and Chesapeake Bay, MD) appeared to be the most similar to MBv200m. A library prepared from coastal California (Scripps Pier, La Jolla) was slightly more distant. In the reciprocal comparison, with MBv200m as the query library, the percentage of sequences hit in the Sargasso Sea library (6.5%) was highest, exceeding that for Mission Bay (3.5%) and Chesapeake Bay (3.9%), but only after normalizing for sequence length. MBv200m was less similar to the viral metagenomes prepared from waters from the Gulf of Mexico, coastal British Columbia, and coastal Arctic Ocean, with the latter being the least similar by both measures.

The similarity between MBv200m and the Chesapeake Bay library was also reflected in a clustering analysis performed in MG-RAST v3 (not shown). MBv200m was most similar to the metagenome prepared from the Chesapeake Bay when clustering based on organism classification frequencies. When clustering was based on functional classifications, MBv200m clustered with metagenomes from Chesapeake Bay, Tampa Bay, and the Sargasso Sea, but was the outlier in that group. Viral metagenomes from the Gulf of Mexico and coastal British Columbia formed a second cluster along with the outlier Arctic Ocean.

## Discussion

Viruses, for the purpose of this investigation, were operationally defined as DNA-containing particles that pass through a 0.2 μm filter, but are retained by a 30 kDa NMWCO membrane and have a buoyant density in the range of ca. 1.3 to 1.5. This is a somewhat restrictive definition that excludes low-density viruses and under-represents or completely excludes very large viruses. Viruses with buoyant densities in CsCl of < 1.3 and > 1.5 have been reported [30], but their contribution to total viral DNA mass in the ocean appears to be very small. In one previous study, all viral DNA detectable on an agarose gel was found in fractions between 1.35 and 1.46 g ml^-1 ^[31]. We found that virtually all of the DNA-containing, virus-sized particles detectable by epifluorescence microscopy in the sample were within a narrower buoyant density range than the known limits for all viruses, and we harvested accordingly.

The virus concentration of our initial sample was not measured, so recovery efficiency cannot be calculated exactly. However, previous determinations of viral abundance at the same station and depth (six samplings between June 1998 and August 1999) ranged from 3.9 to 5.5 × 10^9 ^l^-1 ^(Steward unpublished data). Assuming that our sample fell within this range, we estimate that the final recovery of filtered, concentrated, and CsCl-purified viruses was around 3-4%. Each of the processing steps, and the storage of the concentrate, could have contributed to the loss of viruses, but the yield was not quantified at each step.

Based on the final yield of virus-like particles and the mass of DNA extracted from them, we infer an average DNA content of 42 attograms per virus. The size distribution of virus-like genomes in the final sample was similar to that reported previously from other marine samples. This distribution was not significantly altered even after organic extraction indicating that sample handling and the extraction procedure itself did not cause substantial DNA shearing or any obvious selective loss of DNA from specific viral types.

### Library limitations

With substantial losses of viruses during harvesting, there are likely some biases introduced. Filtration, for example, which was used to remove cells, will also discriminate against viruses with capsid diameters > 0.2 μm. Such large viruses are present in seawater [32-35], but appear to be relatively rare, with the majority of viruses having capsid sizes in the range of 30-100 nm [33]. However, even among viruses with capsids that are < 0.2 μm in diameter, larger viruses and tailed viruses appear to be preferentially lost during filtration [36]. Losses during sample storage were also likely biased, but how and to what degree is unknown. The DNA size distribution among the harvested viruses was similar to the multimodal distributions of virus-like genomes observed previously [31,37] and spanned the sizes of genomes for known bacteriophages and algal viruses [31]. The viral genome size distribution suggests that, even if biased, the library represents a broad spectrum of the viral diversity.

Others have reported difficulty in generating representative libraries of viral DNA by direct cloning [11]. The reasons for this are not clear, but it may be a result of DNA modifications that inhibit ligation or replication of the recombinant plasmid in *E. coli*. We had no difficulties generating a library with unamplified material, and the sequence composition as determined by BLAST was similar to previous studies, all of which used some form of *in vitro *amplification. This indicates that direct cloning of DNA from diverse types of viruses in seawater is feasible. Assuming sufficient material is available for direct cloning, this approach may avoid biases that can sometimes result from amplification. However, if many viruses do indeed have DNA that is not clonable without first being replicated *in vitro*, then the direct cloning approach we employed will necessarily introduce bias as well.

As observed for other viral metagenomic libraries [e.g., [9-11]], the proportion of independent sequences forming apparently legitimate contigs (i.e., those assembled from independent, random fragments) was low. Close inspection of the six putative contigs suggests that even these were most likely assembled from identical clones that appeared twice in the library. In all but one case, the contigs were formed between clones in adjacent wells of a library plate, suggesting possible cross-contamination. And in all cases, the beginning and end position of the clones was quite similar. We therefore found no convincing evidence of there being legitimate contigs, which is not too surprising considering the limited number of sequences comprising the library.

### On the viral nature of the library

Although we specifically targeted the viral fraction of our sample, some of our results suggested that bacterial DNA might be present. For example, we detected weak PCR amplification in our DNA extract with 16S rRNA primers. The single 16S rRNA phylotype detected was not one that had been observed in previous 16S rRNA libraries from Monterey Bay [22]. The close affiliation of this gene with that from a psychrophilic marine bacterial isolate, however, suggests that the sequence may have derived from a legitimate constituent of the Monterey Bay mesopelagic bacterioplankton. The presence of the gene may be due to passage of bacteria or dissolved bacterial DNA through the 0.2 μm filters, it may represent a bacterial DNA fragment in a transducing phage, or it may have been present as a contaminant in our PCR reagents or solutions. No 16S rRNA sequences were found among the viral shotgun library clones sequenced, which was encouraging, but the library is too small relative to the size of a typical bacterial genome for this absence to be informative.

We also found that the majority of BLAST hits with an E-value < 10^-3 ^were not to viruses, but to bacteria, which has been seen in other of viral metagenomes [9,10]. In some libraries, hits to viral sequences exceeded those to bacterial sequences [11], but hits to non-viral sequences are always common [2]. Although this could reflect bacterial contamination, some have speculated gene-transfer agents (GTAs) might be responsible [7]. GTAs are virus-like particles carrying random fragments of DNA sampled from the host from which they derive [38]. We cannot conclusively rule out the presence of either bacterial contamination or GTAs as source of bacterial signal in our library, but below we discuss evidence that suggests viral DNA dominates our library.

We did not detect bacterial cells among the viruses harvested from the CsCl gradient, which suggests that contamination with cells from the original sample, if present, was low. Furthermore, our empirical estimate of DNA content per recovered virus (4.2 × 10^-17 ^g virus^-1^) is somewhat lower than a previously reported average of 5.5 × 10^-17 ^g virus^-1 ^for a variety of marine habitats [31], but is within the range of values from which that average was calculated. This suggests that the number of virus-like particles extracted can account for the majority of the DNA. If the viral DNA is dominated by double-stranded genomes, as was recently observed in Chesapeake Bay [12], the calculated DNA content per virus implies an average viral genome size of 38 kb. With 390 kb of total sequence analyzed from our library, a single-copy viral gene could appear up to about ten times if all the DNA is of viral origin, but only if present and recognizable in every virus. Most functional categories of viral genes were present fewer than ten times, but there were nine clones with a top hit to phage terminases. This complementary analysis is also consistent with the majority of DNA being derived from viruses, and bacteriophages in particular, rather than GTAs.

If our library is dominated by viral DNA, then the predominance of hits to bacteria and microbial metagenomes, rather than to viruses and viral metagenomes, might be best explained as an artifact of biased sequence representation in GenBank and the presence of undocumented viral sequences within bacterial genome sequences. It has been noted that even genome sequences from purified viral isolates can generate many top BLAST hits to bacteria [11]. The dramatic increase in the recognition of hits to phages in the latest version of MG-RAST suggests that this bias is being reduced as more viral sequences become available. Our manual annotation found many more significant hits to viruses, however, suggesting that such automated pipelines still have limitations.

Microbial metagenomes include many viral sequences that may derive from the capture of free or adsorbed viruses, prophages, and infected cells [8,39]. Identifying the viral sequences in the large background of cell-derived sequences in a microbial metagenome is challenging and requires a conservative approach [8]. Since it is impossible to prepare a microbial metagenome free of viruses, but viruses can be prepared virtually cell-free, analyses of targeted viral metagenomes will be helpful in determining the likely sources of DNA sequences in microbial metagenomes.

### Sequence analysis

Since our source material was DNA from what appears to have been highly purified virus-like particles, the break point in the hit distribution is a useful empirical indicator of a threshold beyond which the quality of hits quickly degrades. In our analysis, the E-value threshold of 10^-3 ^appears to be slightly conservative for blastx and slightly generous for tblastx, but seems to be a reasonable compromise for general application. A similar analysis using E-value bins rather than a running threshold provided further empirical support for the use of an E-value threshold of 10^-3^. We therefore adopted this commonly used threshold [9,11] when we designated BLAST matches as "signifcant hits". The exception was for the inter-library comparisons where we employed a more restrictive criterion of E ≤ 10^-5 ^also used by others [10]. A comparison of the hit distributions indicates that blastx was generally more useful than tblastx for identifying meaningful matches in the GenBank databases. However, tblastx did identify some matches to viruses that were missed by blastx, suggesting that using both algorithms, rather than relying on one, can be beneficial. In many cases, the top hit was not very informative. Our use of a keyword search of multiple databases (especially Pfam and KEGG) was helpful in identifying hits that were significant, but lower scoring, matches to sequences with putative viral functions.

Although our sample was collected below the euphotic zone, many of the virus hits were to viruses known to infect phytoplankton (cyanophages and phycodnaviruses). This may reflect the fact that phytoplankton (and their viruses) are continually transported into deeper waters through association with sinking particulates [40] or via grazing by vertically migrating zooplankton [41], but could also reflect the existence of genetically similar viruses infecting photosynthetic and non-photosynthetic microorganisms. The depth at which we sampled was previously found to be the depth at which marine crenarchaea reach their peak abundance in Monterey Bay at about 20% of the total prokaryotes [42,43]. Despite this, top hits to archaeal genes comprised only 3% of the total and there were no hits to phages known to infect archaea. This most likely reflects the fact that cultured representatives of the marine planktonic archaea are still scarce [44]. These marine archaea are divergent from the better studied thermophilic and methanogenic representatives [45,46] and viruses infecting them have not yet been isolated or described.

The distribution of hits in our library is similar to previous viral metagenomes [9,11] in that hits to bacteriophages were more common than to eukaryotic viruses. This is consistent with the other indirect evidence that bacteriophages dominate the planktonic viral assemblages [reviewed in [47]]. As found for the Mission Bay library [11], genes involved in DNA modification, specifically terminases, were the most common viral hits in our library, followed by hits to viral structural genes. In other libraries (e.g., Scripps Pier, Chesapeake Bay), structural genes were the most common [9,11].

### Library Comparisons

The relative greater similarity between the Monterey Bay library and the two viral metagenomes from other bays suggests that water from these similar types of eutrophic embayments have more similar communities. We note, however, that the percentage of sequences in the Mission Bay and Chesapeake Bay libraries that had a significant match with any sequence in MBv200m was still relatively small (≤ 6% even when not length-normalized). This is not too surprising since Mission Bay, Chesapeake Bay, and Monterey Bay are quite different in their physiography and hydrography and represent coastal waters of two different oceans. In particular, the station sampled in Monterey Bay is more oceanic and the sample was collected at much greater depth than either the Mission Bay or Chesapeake Bay libraries. The low coverage of these three libraries (873 to 5641 sequences) is also likely inadequate to properly capture the range of diversity present at each site.

A possible artifact in similarity analyses may result from differences in the read lengths of the libraries [7]. The three most similar metagenomes consisted of longer reads by Sanger sequencing (mean read lengths from 607 to 695 bp), while the four more distant libraries were all composed of shorter reads (mean read lengths ≤ 104 bp). To compensate for this, we normalized the number of BLAST hits by read length. This correction decreased the distance measures between our library and those generated by next-generation sequencing platforms, as expected, but in most cases the rank order was unchanged. One exception was the Sargasso Sea library, which had the third highest percentage of sequences with a significant match to MBv200m prior to length-normalization (data not shown), but the highest after. Many other differences in the way viruses were harvested and purified, the manner in which DNA was prepared for sequencing, and the sequencing methods used, preclude us from drawing any meaningful ecological inferences from these inter-comparisons. Nevertheless, the comparisons provide some sense of the differences in the sequence content of the libraries. We note that the Arctic library is by far the most distant from our library, and was also found to be the most distant from three other samples to which it was originally compared [10].

## Conclusion

The viral metagenome described in this paper is the first to be reported from a single depth below the euphotic zone in the ocean and with no amplification prior to cloning. Our data suggest it is possible to clone viral DNA with no *in vitro *amplification, although, as with any of the preparation methods currently in use, there may be biases. Indirect evidence suggests that, although most of the top BLAST hits were to sequences annotated as bacterial or to microbial metagenomes, most of the sequences in our library are probably of viral origin. This means that the majority of viral sequences in microbial metagenomes will be unrecognizable as such. Analyses of virus-targeted metagenomes, like the one reported here, are therefore a valuable complement to studies of microbial metagenomes and may assist in discriminating the likely source of novel sequences.

## Competing interests

The authors declare that they have no competing interests.

## Authors' contributions

CMP collected and purified the viruses, constructed the library, conducted the sequencing, co-wrote the manuscript and helped prepare figures. GFS provided advice on sample preparation, assisted in clone picking, processed and analyzed the sequence data, prepared the figures, and co-wrote the manuscript. Both author's read and approved the final manuscript.
